# The Odor Characterizations and Reproductions in Machine Olfactions: A Review

**DOI:** 10.3390/s18072329

**Published:** 2018-07-18

**Authors:** Tengteng Wen, Dehan Luo, Jiafeng He, Kai Mei

**Affiliations:** School of Information Engineering, Guangdong University of Technology, Guangzhou 510006, China; wentt.cn@outlook.com (T.W.); jfhe@gdut.edu.cn (J.H.); mkai2017@outlook.com (K.M.)

**Keywords:** machine olfaction, electronic noses, odor characterizations, odor reproductions, olfactory displays

## Abstract

Machine olfaction is a novel technology and has been developed for many years. The electronic nose with an array of gas sensors, a crucial application form of the machine olfaction, is capable of sensing not only odorous compounds, but also odorless chemicals. Because of its fast response, mobility and easy of use, the electronic nose has been applied to scientific and commercial uses such as environment monitoring and food processing inspection. Additionally, odor characterization and reproduction are the two novel parts of machine olfaction, which extend the field of machine olfaction. Odor characterization is the technique that characterizes odorants as some form of general odor information. At present, there have already been odor characterizations by means of the electronic nose. Odor reproduction is the technique that re-produces an odor by some form of general odor information and displays the odor by the olfactory display. It enhances the human ability of controlling odors just as the control of light and voice. In analogy to visual and auditory display technologies, is it possible that the olfactory display will be used in our daily life? There have already been some efforts toward odor reproduction and olfactory displays.

## 1. Introduction

The earliest research on a machine olfaction system can be dated to 1961 [1]. The electronic nose is comprised of an array of gas sensors and is capable of sensing both odorous and odorless volatile compounds. Up till now, machine olfaction techniques have been applied to many different fields such as food processing [2,3], alcohol producing [4,5], environmental detections [6,7], Chinese herb classifications [8], etc. For instance, an electronic nose can be applied to monitor the quality of wine in the fermentation stages [9]. An electronic nose can be set up near a waste yard or outfall, so that it is capable of monitoring pollution gases online. Compared to other detecting instruments, electronic noses have several advantages. Firstly, gas sensors are capable of sensing odorants and odorless volatile chemicals, but we human beings are only able to percept odorants. Currently, some product quality inspections are being implemented by sniffers who are easily affected by internal factors such as emotions and external factors such as linguistic interferences. Inspection by means of an electronic nose is a more reliable and objective method. Secondly, the electronic nose is smaller in size and has higher mobility. This makes the electronic nose a suitable instrument to detect the quality of raw materials or products by sensing their volatile chemicals on the production line. Thirdly, the speed of detection by an electronic nose is much faster because of a lower measuring time compared to other instruments such as a mass spectrometry. Besides, the mobility of an electronic nose makes it possible to set up real-time and on-site detection, which reduce the long travel from the sampling site to a laboratory. However, the electronic nose has some bottlenecks. For instance, the performance in an open environment is unsatisfactory because of the turbulence of airflow. The replacement of a gas sensor in the array leads to the retraining of the system.

The term electronic nose, first defined by Prof. J. W. Gardner in 1994, is restricted to an intelligent odor sensory device that consists of an array of gas sensors and an intelligent recognition module [10]. Metal-oxide-based gas sensor arrays are widely used in electronic noses for due to their better performances. However, a metal-oxide-based gas sensor array in an electronic nose still has some bottlenecks, such as long-term drifts, lower reproducibility and cross-sensitivity. The price of some models is relatively high, which may limit their commercial uses. There have been already some efforts to reduce these negative effects by researchers [11,12,13,14,15,16,17].

**Figure 1:**
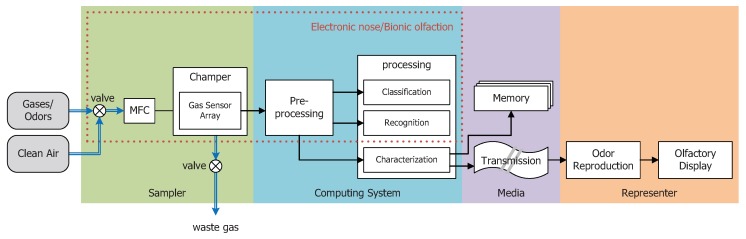
A typical scheme of machine olfaction. MFC, mass flow control.

Nowadays, the terms machine olfaction and electronic nose are confused in many papers to some extent. In this paper, as shown in Figure 1, we would like to emphasize the term machine olfaction, which consists of several parts:

1. a sampler,
2. a computing system,
3. a medium and
4. a representor.

Figure 1 depicts a general system of machine olfaction. The sampler contains a chamber with an array of gas sensors, which transduce chemical features of gases or odorants into electronic signals. A sampler also consists of a mass flow control (MFC) unit for target gases or clean air inflows and exhaust. The computing system is comprised of a pre-processing block for the background effect suppression, drift compensation, robustness improvement, etc. The processing block is used for the calculation of classification, recognition and odor characterization. After that, the odor characteristics extracted by the computing system can be stored in some memory space or can be transmitted to a remote place for odor representing. The odor representor implements the odor reproduction and display. We follow the definition of the term electronic nose defined by Prof. Gardner. The red-dot frame in Figure 1 depicts a typical model of the electronic nose. Other instruments such as mass spectrometers [18], gas chromatographs [19], ion mobility spectrometers [20,21] or surface acoustic wave resonators [22] are regarded as machine olfaction apparatuses of other forms to some extent. The paper focuses on the electronic nose with an array of gas sensors for odor characterizations.

**Figure 2:**
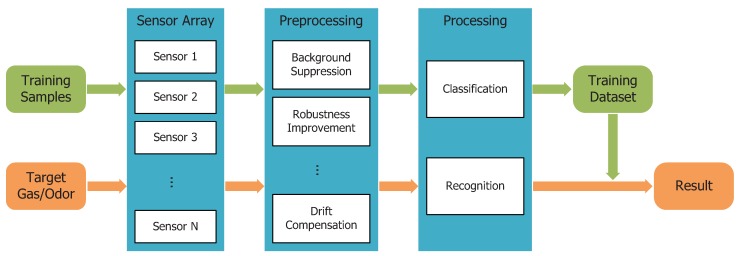
A typical scheme of machine olfaction.

Figure 2 describes a general idea of the gas/odor sensing and recognition mechanism. Training samples are carefully selected, trained and saved. After that, target gases or odors can be input and recognized by the system with the trained dataset. Up till now, the majority of research on machine olfaction has focused on gas/odor classifications or recognitions. The research on odor characterizations is lacking in attention. R. Axel and L. B. Buck, who were awarded the Nobel Prize in Physiology or Medicine 2004, discovered the mechanism for how we human beings discriminate scents [23]. By analogy with machine vision that colors can be divided into red, green and blue, is there a theory that is able to describe odors in a more general way? Unfortunately, there is not universal agreement about such a general theory that would be sufficient to depict the relation of odorants and odor qualities. This limits the development of the qualification and quantification of odor properties. Besides, the performance of single gas sensors or a sensor array are unsatisfactory for sensing and discriminating such a large number of different odors in nature.

Olfaction is another significant sensory perceptual system following vision and audition. Nowadays, scent is considered to be another dimension that is capable of the enhancement of multimedia systems. For instance, presenting scents would be a novel way to expand the imagination of a movie director. Reproducing smells in front of screens when users go shopping on e-markets would improve the online shopping experience. Virtual reality is another field to which odor reproduction could be applied. In Figure 1, the odor presenter (orange area) is the system for reproducing and presenting odors from some odor characteristics encoded from the sampling system (the characterization block in the blue area). The odor reproduction block conducts decoding of odor characteristic codes and offers a recipe to re-generate odor. The olfactory display block is an apparatus to display odors by some carefully designed machines. Currently, a number of research works on olfactory displays have been reported in recent decades, while quite a few studies have focused on odor reproductions.

The paper reviews the odor characterization by an electronic nose with an array of gas sensors and odor reproduction in recent decades. In the following paragraphs, current measurements of odor concentrations and cluster discriminations will be illustrated in the second section. In the third section, the progress of olfactory displays in the last decade will be demonstrated. After that, a discussion about the combination of odor characteristics and odor reproductions will be made.

## 2. General Odor Characterization

Intensity and quality are two properties that odorant chemicals have that influence the olfactory perception [24]. Odorant concentration is a significant property that depicts the intensity of odors and influences the olfactory perception. Gas concentration measurement has been applied to many fields such as industry [25], agriculture [26] and environment [27,28]. Since gas concentration measurements have a referential value to odor intensity (odorant concentration) measurements, we reviewed both gas and odorant concentration measurements at the same time. Besides, the quality of an odor depends on the chemical features of the odorant and the olfactory mechanism of odor perception. Some efforts toward odor clustering using an electronic nose have been reported.

### 2.1. Measurement of Gas/Odorant Concentrations

Electronic noses perform decently in qualitative analysis, but not in quantitative analysis. An array with gas sensors carefully selected is the fundamental basis for odorant concentration measurement. Different odorants have different response patterns on the sensor array. The relation of concentration and the response pattern is fitted by appropriate methods, such as linear regression or artificial neural networks (ANNs). Overfitting or underfitting will lead to the failure of odor concentration quantification. Efforts were made by some researchers and will be reviewed in the following paragraphs.

K. C. Persaud et al. investigated 10 odorants in three pigs fed using an array with 20 polymer-based sensors [29]. The base-resistance ΔR/R in air was detected for over one week. Each of 20 sensor responses for ethanol showed almost a linear relation to concentration, while, for methanol, a nonlinear relation was depicted. T. H. Misselbrook et al. set up three experiments in a slurry and utilized electronic noses (Odormapper and AromaScan) to investigate odor concentrations [30]. Sensor response was averaged and fitted to odor concentration by a linear regression. Parallel regression analysis was implemented, and the analysis showed there was no significant difference between the relationships for the individual experiments. It revealed a relatively linear relationships between odor concentration and average sensor response.

G. Qu et al. designed an experiment to measure concentration by an electronic nose [31]. The comparative experiment involved 44 persons and was followed by the draft CEN standards. An electronic nose with an array of 32 sensors was used. Principal component analysis (PCA) was first implemented to remove insensitive inputs from 34 inputs, which consisted of 32 sensor responses and two humidity data (odor sample and reference air). After that, the adaptive logic network (ALN), which is one of artificial neural network algorithms, was implemented. The result demonstrated that a well-trained ANN combined with an electronic nose was capable of measuring odor concentration with a relatively low error.

**Figure 3:**
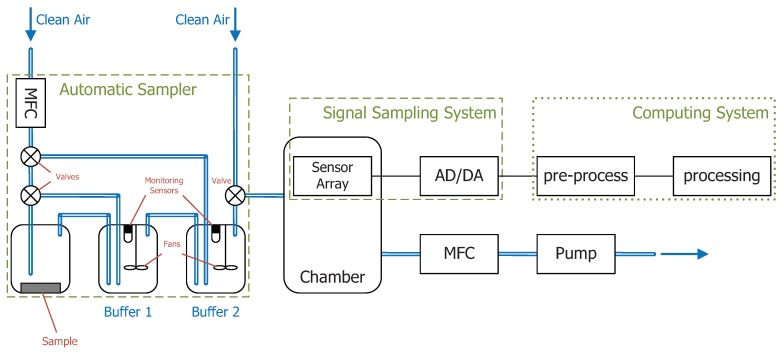
Gas/odor concentration measurement system with an automatic sampler.

In 2001, T. Maekawa et al. designed a complete odor identification system, which integrated an automatic sampler with hardware and software [32]. For the purpose of speeding up the measurement, an automatic sampler composed of three gas chambers in series connection was designed, as Figure 3 shows. The first chamber in the left-most part is used to place the sample. The other two chambers (Buffer 1 and Buffer 2) are used for the concentration adjustment so that the sample’s concentration in Buffer 2 can be tuned to the present value. Clean air was controlled by a mass flow controller, and two electromagnetic valves in cascade connection were used to control the flow direction. The gas flow released from the second chamber is conveyed to an array of eight metal-oxide-based sensors. Responses from the sensor array were transmitted to a personal computer for signal processing. PCA, cluster analysis and the ANN algorithm for odor identification were implemented. The result revealed that the automatic sampler is capable of stabilizing sensory datasets for the subsequent analysis. Although this paper did not focus on the gas concentration detection, the hardware design for adjusting and controlling the concentration automatically offers us a means of measuring and investigating concentration by an electronic nose with a sample structure.

A metal-oxide-based gas sensor array was developed and a new method was proposed for the concentration measurement of H${}_{2}$S and NO${}_{2}$ by using discriminant factorial analysis [33,34]. The result indicated a good qualitative discrimination of H${}_{2}$S, NO${}_{2}$ and their mixture. The quantitative identification of the gas concentrations was not as good as the qualitative identification, though the NO${}_{2}$ quantification with 7 ppm H${}_{2}$S as background gas was excellent.

D. Gao and W. Chen proposed function approximation model ensembles and combination strategies using multivariate logarithmic regressions (MVLR), quadratic multivariate logarithmic regression (QMVLR), multilayer perceptron (MLP) with sigmoid activation functions and support vector machine (SVM) with radial basis function kernels [35]. In order to validate their ideas, they developed an electronic nose with an array of 16 metal-oxide sensors fabricated by Figaro Engineering Inc., Tokyo, Japan. The experimental result revealed that the combination strategies were quite effective.

S. D. Vitoet al. developed an array of five hybrid room temperature operating chemo-resistive sensors for continuous sampling and estimations of gas concentrations [36]. Two tapped delay fusion architectures were proposed in their research. One is a tapped delay neural network (TDNN) in back propagation architectures, and the other one is a combination of tapped delay lines and support vector machine in regression mode (TD-SVR). A continuous test of which various concentration combinations of NO${}_{2}$, NH${}_{3}$ along with different relative humidity were set in each sampling time period was conducted. Performances were calculated and evaluated by the mean absolute estimation errors. Although both architectures were considered to be satisfactory, TD-SVR performed better than TDNN.

G. Hodon et al. investigated odor intensities of several odorants using two commercial electronic noses and an experiment-made one, the three electronic noses of which were comprised of different types of sensors [37]. They did a comparative analysis of the linear relationship between odor intensity and average sensor response by means of linear regression analysis and artificial neural networks (ANN). The result indicated that ANN presented a much better correlation between measured odor intensity and calculated odor intensity. They also suggested that a sufficient number of sensors with enough diversity is an alternative to enhance the capability of odor intensity determination.

J. G. Monroy et al. proposed a gas concentration quantitative method for an open sampling circumstance in 2013 [38]. A single sensor or an array of metal oxide was used for analysis, and the measurement system was referred to as an open sampling system. The greatest uncertainty of sampling in an exposed condition is thought to be the turbulence of airflow, which will impact the volatile compounds in contact with the sensors. They investigated and evaluated the performances of either single sensors and a sensor array by the continuous sampling in an open circumstance. Gaussian process regression was used for gas concentration estimation, and automatic relevance determination was used for feature selection. In order to evaluate the performance of the three models and each sensor, root mean squared errors (RMSE) and negative log predictive density (NLPD) were calculated.

(1) $$RMSE=\sqrt{\frac{1}{n}\sum_{i=1}^{n}\left(c_{i}-{\bar{c}}_{*i}\right)}$$

(2) $$NLPD=-\frac{1}{n}\sum_{i=1}^{n}\log\left(p\left(c_{i}|r_{i}\right)\right)$$

$c_{i}$ is the actual gas concentration and ${\bar{c}}_{*i}$ is a mean value of the estimated concentration of the *i*-th sample. The term *n* is the number of training samples, and $r_{i}$ represents the response of each sensor. The evaluation results of single sensors indicated that different single sensors perform differently, but each single sensor performs similarly in three regression models.

P. Pławiak and K. Rzecki compared and evaluated several artificial neural networks (ANNs) and hybrid systems for predicting the concentration of phenol in 2015 [39]. Samples were collected by a commercial electronic nose-FOX 4000 manufactured by Alpha M.O.S. company (Toulouse, France). The basis of the accuracy and complexity criteria are two aspects for the comparison and evaluation of those methods. The result demonstrated that the performance of the radial neural networks is better than the other methods.

L. Eusebio et al. proposed an electronic nose testing procedure [27]. For the purpose of the minimum performance requirement for environmental odor monitoring, a criteria with three levels was set: (i) invariability of responses to variable atmospheric conditions; (ii) detection limit; (iii) classification accuracy. The experiment was equipped with two experiment-made electronic noses for environmental odor sampling and monitoring [40]. Concentrations of five target odorant samples were calculated by semi-empirical equations with compound-specific coefficients, and these equations can be found in [41]. Temperature and relative humidity (RH) were the two parameters considered. The results revealed that the performances of the tested electronic noses were decent at 70% RH and at 23 ${}^{{}^{\circ}}$C.

Table 1 demonstrates a summary of gas/odor concentration estimation research works. The research of gas/odor concentration estimation by means of the electronic nose with a gas sensor array is still in the process of scientific research. Only a few of the research works were applied to food production and environmental detection. Commercial electronic noses were applied to a proportion of research works, but a number of experiment-made (EM) electronic noses were used in some researches. Most of the research works employed metal-oxide-based (MOX) or polymer-based sensor arrays. The research of gas/odor concentration estimation is in the primary stage, and it is worth deeper research.

### 2.2. Separation into Molecular Features

There are a few works about how to distinguish molecular features of gases by means of electronic noses. In order to separate odorants, some efforts by means of integrating an external device into the sensory system were conducted. M. Imahashi et al. [42] designed an odor separating system to mimic the biological olfactory system. In order to imitate the mechanism of biological odorant perception, the system contains an adsorbing and separating module that consists of several such cells to adsorb odorants by different electrical polarities. A sensory module consists of several sensor cells to measure odorants. Odorants will be first filtered and adsorbed by adsorbates in the adsorbing and separating module. After that, these adsorbed odorants will be released by micro ceramic heaters controlled by current and will be flowed into the sensory module for detection. In this experiment, Imahashi had successfully measured odorant in four clusters A–D. The molecular imprinting technique (MIT) involves the preparation of a polymer with selective recognition sites for a specific molecule. M. Imahashi and K. Hayashi developed a nanofilter that is capable of discriminating complex molecular profiles and molecular functional groups [43]. In their works, they successfully discriminated propanoic acid, hexanoic acid, octanoic acid, heptanal, 3-octanone, heptanal and heptanoic acid.

## 3. Odor Reproduction

The olfactory display is a system of representing odors, which is comprised of an odorous gas generator, a gas blender, a gas releaser along with an embedded control component. The odor reproduction comprises an olfactory display apparatus and a set of recipes for producing odors by a group of aroma materials. We searched four key words, namely “odor display”, “odor reproduction”, “olfactory display” and “odor generator”, on Google Scholar (allintitle: “odor display” OR “odor reproduction” OR “olfactory display” OR “odor generator”) and the Web of Science (TOPIC: (“odor display”) OR TOPIC: (“odor reproduction”) OR TOPIC: (“olfactory display”) OR TOPIC: (“odor generator”) Timespan: 2009–2018. Search language = Auto). The number of literature works about this field is surprisingly low. On Google Scholar, there are only 63 papers including journals and conference papers, while there are only 37 papers on the Web of Science in the last ten years (2009–2018) (searched on 23 April 2018). In this section, we will review the development of the odor reproduction/display in recent ten years.

### Olfactory Displays

There had been already some efforts to enhance the performance of odorant releasing. The most significant problem of an olfactory display is the rate of vaporization of aroma materials. Since aroma materials can be either creams or liquids, the means of vaporization should be taken into account. Thermalizing the aroma material, increasing the airflow or vibrating the aroma liquid are common methods of vaporization. Fans also play a key role in conveying odorant to users’ noses. An alternative way is based on ultrasound to control the direction of odorant airflow.

There are several methods to accelerate producing scents, which had been summarized by Y. Yanagida in 2012 [44]. Firstly, vaporization of perfume sources can be accelerated by increasing the airflow rate at the liquid surface, bubbling the liquid and blowing the gel or porous material in a hermetic vessel. Secondly, heating is one of the traditional ways of accelerating the production process. Thirdly, there are several techniques of atomization to speed up the production of odors, which are: (i) sprayer; (ii) air-flow-based atomizer consisting of two nozzles, one of which is used for blowing fresh air and the other for spraying the perfume liquid; (iii) ultrasonic wave device or surface acoustic wave (SAW) device; (iv) ink-jet head, which is widely used in ink-jet printers.

Thermalizing is one of the common vaporization methods used in some olfactory displays. D. W. Kim et al. developed an olfactory display device consisting of a 3 × 5 array of thermal-based metal disk-shaped copper plates [45]. Since the vaporization method used in this device was heating, the power consumption was relatively high, and the response rate of releasing the scent was low. The aroma sources should be thermalized carefully, otherwise scorching may happen.

Fans are important components in most olfactory displays. H. Matsukura et al. combined an image screen with two bars attached on both sides of the screen [46]. The airflow directions of the two bars were opposite and parallel on the screen. When the screen presented some typical objects, the two bars released the target odor generated by an external device, the odor vapor of which was generated by bubbling air at a flow rate of 500 mL/min through a liquid perfume and transported to the display bars through a tube. The interval time between odor releasing and recognition by a user was unsatisfactory. Herrera, Howell and their team developed a simple and cost-effective olfactory display, in which a fan was used to speed up the release of an odor [47,48].

A combination of heating and fan is a more common way of designing a thermal-based olfactory display. A thermal-based odor display was designed by J. A. Covington et al. [49]. The device consisted of a metal-oxide gas sensor for monitoring and controlling the release of the scent. LabVIEW custom software, National Instruments Corporation, Austin, USA was designed for selecting the scent and configuring the intensity and airflow. It was called the “aroma generator”. The metal-oxide gas sensor was used for monitoring the volume of odorous gas. The thermal-based wearable odor necklace was capable of releasing six different scents when users’ phones had incoming calls in [50].

Ink-jet technology, a very common technology used in printers, had been applied to dropping aroma liquid. S. Sugimoto et al. developed an olfactory display for which ink-jet components were used to jet aroma liquid in a tank [51]. The jetted-out liquid was heated then blown out the device by a fan. In order to switch different scents in a short time, A. Kadowaki et al. proposed a pulse ejection method capable of reducing the volume of scent release and increasing the ability of switching to different scents as soon as possible [52].

Aerosol sprays are commonly-used articles in our daily life. K. Sakamoto et al. designed an olfactory display apparatus using an automatic spray controller, and the display apparatus was applied for memory therapy [53]. Since the control of spray motion is conducted by mechanical devices, it is inevitable that the complexity of such a controller is high.

The surface acoustic wave (SAW), which is a nanoscale amplitude wave traveling along the surface of a material, was first reported by Lord Rayleigh in 1885 [54]. The nanoscale wave traveling along a piezoelectric film is usually generated by an interdigital transducer (IDT). Several factors have impacts on the performance of such SAW devices: the substrate depth, the surface tension, the frequency of the surface waves and liquid properties (density, shear viscosity, the characteristic height and length scales) [55]. Y. Ariyakul et al. developed an olfactory display that consisted of an electroosmotic (EO) pump and a surface acoustic wave (SAW) component [56]. The volume of perfume liquid used in each display stage was controlled by the EO pump, and the atomization rate was controlled by IDT, which consisted of two interlocking comb-shaped arrays of metallic electrodes. K. Hashimoto and T. Nakamoto designed a wearable olfactory display using a stabilized SAW atomizer [57,58]. Since the viscosity of the liquid influences the atomization capability and efficiency, they used ethanol to dilute some high-viscosity perfumes. An oscillator of 400 MHz was converted to approximately 61 MHz by phase locking loop (PLL), and a direct digital synthesizer (DDS) was used to adjust the frequencies of the output signal in an Altera FPGA chip. The output wave was amplified by an RF amplifier, which was used to drive the SAW device. The FPGA chip was used to set the driving signal waveform for the control of micropumps for injecting perfume liquid. E. Matsuura et al. developed an olfactory display consisting of four piezoelectric ink-jet components for dispensing aroma liquids. A fan with 10 rotation speeds was at the back of the device and controlled by software [59]. The purpose of the device was for medical testing. S. H. Abid et al. proposed a solution of enhancing the performance of micro-porous piezoelectric film by covering it with steel mesh [60]. They also proposed bitmaps for scent generation, which were inserted into video files. When a bitmap was decoded by a video decoder, the olfactory display would generate the specified odor as soon as possible. A SAW-based olfactory display integrated into a head mount display (HMD) was proposed by S. Itou et al. [61]. Since the hydrophobicity on the surface of the SAW device was supposed to be an reasonable characteristic to enhance the capability of atomization, H. Li et al. proposed to use an amorphous fluoropolymer film with a 400 nm-thick coating on the SAW substrate in their other work [62].

Table 2 summarizes and compares olfactory displays in several aspects: the vaporization types (Type), the response rate (Resp. Rate) of generating an odor, the noise levels (Noise), the power consumption (PC), the number of odors (NOs) that a display was able to generate, the forms (wearable or projected on desk) and the controller complexity of the display. The SAW-based olfactory display generates scents faster with a relatively low noise level. In addition, the SAW-based olfactory display has a lower complexity in designing the structure. The exquisite ink-jet device, which is widely used in ink-jet printers, was capable of controlling the liquid volume precisely, and the number of different aroma sources was relatively higher than other vaporization types.

A number of applications had been reported in the last ten years. For the purpose of increasing the effect of an advertisement, C. Pornpanomchai et al. proposed a schematic of advertisement system along with a simple odor display [63]. The system released one of four odors when the watermark, which was able to display four colors, displayed a specific color. T. Nakamoto et al. also designed an odor display prototype comprised of an odor generator and an odor recipe configuration desktop software [64]. The system is capable of presenting a PowerPoint slide synchronized with a concentration-configurable target odor.

Virtual reality (VR) technology had been developed in the last six decades, and a number of efforts had already been made to add olfaction as another sense in VR. Two forms are most commonly used in current VR technology, which are headsets [57,58] or multi-projected environments [46,47,48,53,61,65,66]. Considering the size of an olfactory display, there have been many efforts to reduce the size of display structure and increase the response rate of the display.

Some efforts were concentrated on medical uses [53,59]. Olfactory display necklaces were another form, in which the necklace would release odors in some specified circumstance such as receiving a message on a phone [50].

## 4. Discussion

### 4.1. Is There a Sufficient Interpretation of the Feature-Odor Relation?

Odorants are odorous chemicals, and the molecular weights of such chemicals are less than 300 Da. It is widely acknowledged that one odorant is detected by a cluster of different olfactory receptors (ORs), and one OR is capable of detecting a group of different odorants. There are about 1000 genes in the olfactory gene family, so humans may possess up to 1000 types of odorant receptors (OR) [67]. It is acknowledged that a scent with one or a group of odorants is perceived by a specific group of different olfactory receptors [68,69,70,71,72,73,74,75].

It was estimated that humans are capable of discriminating over one trillion olfactory stimuli [76], so categorization and simplification of odorous compounds are necessary in odor reproduction. Investigations indicate that different odorous stimuli activate distinct patterns of odorant responses and a significant amount of crossover activation among odorants with similar chemical features [75]. Some evidence shows that ORs are partially possibly activated by the overall steriochemical structure of the hydrocarbon chain and the type and position of the functional groups that an odorous molecule possesses [71,72]. In other words, molecular profiles and polar functional groups may be two main features that influence the discrimination of odors. Two main theories about the structure-odor relations had been proposed for decades, which are steriochemical theory and the vibrational theory of olfaction, respectively. Amoore postulated the “steriochemical theory”, which relates odor quality to molecular shape and depicts the concept primary odors [77]. In analogy to primary colors, seven odors were suggested as primary scents, which are “musky”, “putrid”, “pungent”, “camphoraceous”, “ethereal”, “floral” and “pepperminty”, respectively [78]. It was postulated that other odors are combinations of two or more primary odors. The vibrational theory of olfaction (VTO) was first proposed by Dyson [79], later extended by Wright [80,81]. It suggests that odors correlate with vibrational spectra to a certain extent. After a long time of stillness, Turin reincarnated a new version of VTO: that the inelastic electron tunneling spectra of odorant molecules correlates with odors [82]. In VTO, scents are originated from the electron vibrations of odorous molecules, and the odor quality is related to the frequency of vibration. It was assumed that olfactory receptors accept the vibration energy from molecules, then generate and transmit odor signals to the cortex. The two theories illustrated above succeeded in describing the structure-odor relation of some chemical compounds. However, both failed for some exceptions of odorous compounds. Accordingly, there has not been a universal agreement about the mechanism for how we human beings perceive odors yet.

### 4.2. Olfactory Display

Perfumes are used in our daily life world wide. In addition, adding the olfactory dimension to a multimedia system is a way to enhance the experience of multimedia entertainment. Several specifications of the olfactory display should be considered.

Firstly, unlike the propagation speed of light and sound, the propagation rate of odorants is much slower. Therefore, the fast response of releasing a scent should be considered. For instance, a multimedia system with an olfactory display should be able to represent a scent synchronized with a segment of a movie displaying on a screen so that a director would be able to present the scene more realistically and powerfully. The faster the response of an olfactory display apparatus, the bigger the role the olfactory display would play in a multimedia system. In order to speed up the propagation of odorants, one or more fans are applied in many current olfactory displays. However, the period of the slow startup of the fan rotation impedes the fast-response enhancement of an olfactory display. Moreover, the high rotational speed of a fan inevitably generates much noise, which is unacceptable. Although fans were used for controlling the airflow direction in many research works, other techniques such as ultrasound have come into sight. An ultrasound array to control the spatial distribution of odorant airflow was proposed by K. Hasegawa et al. [83]. A Kinect V2 was used to track the user’s face so that the ultrasonic phased array was able to redirect odorants in the air to the user. The ultrasound phased transducer array is capable of generating a virtual conical source to create a Bessel beam so that the airborne delivery happens.

Secondly, it is necessary to investigate the odorant airflow because it is a way to enhance the speed of propagation and reduce the dosage of aroma materials. Odorant airflow plays an important role in the efficiency of an olfactory display. The spatial spot at a user’s nose is the spot that must be investigated. An olfactory simulation system that consisted of an odor display and a computational fluid dynamics (CFD) simulation subsystem was proposed by H. Matsukura et al. [65,84]. The aim of their works was that the system was capable of reproducing the scene simulated by CFD by controlling the airflow-generated odor display. They investigated that the odor concentration at the spatial point where the user perceives the odor is varied by the user, since human body temperature brings the odor vapor along the body to the nose [85]. H. Ishida et al. also proposed a method based on computational fluid dynamics (CFD) simulations to measure the distribution of odor concentration [86]. The path of odorant airflow from an olfactory display to a human’s nose can be calculated so that the interval time between when a scent is released and when human noses perceive it can be calculated. The gas outlet set near the nostril directly is a method for wearable devices, but it is not a reasonable solution for desktop olfactory displays.

Furthermore, different ways of vaporization have an impact on the controller complexity, as higher controller complexity will lead to a bigger size of an olfactory display and a more complex control mechanism. Thermalizing is a commonly-used scheme to release scents from aroma gels, but it is easy to scorch aroma gels. Therefore, a temperature control loop for preventing scorching is indispensable. Sprayer-based olfactory displays have a complex mechanical controller system, and this may be not a good way for designing an olfactory display. The piezoelectric-based olfactory display is simple in structure, and the control system is simpler than other prototypes.

### 4.3. Odor Reproduction Based on Odor Characteristics

Perfumery, the art of producing scents, has a long history of development. Nevertheless, odor reproduction is not identical to perfumery. Odor reproduction is a technology to reproduce scents that are captured from the scene by some odor sensory systems. Up till now, there have only been a few research works on odor reproductions due to the ambiguity of the olfactory perception mechanism. As illustrated above, both the steriochemical theory and VTO successfully describe the odor-structure relationship to some extent, but both theories fail with respect to some exceptions. A recent investigation considering biological response and applying a medicinal approach was reported [75]. The experimental results indicated that the odor discriminations by human beings are more likely determined by olfactory receptors, rather than odorant chemical features. A comparison of two odorant classifications was conducted, and the classifications were based on odorant chemical descriptors and responsive olfactory sensory neurons, respectively. The result revealed that the relative position of the carbonyl group may be the significant factor for how olfactory receptors recognize odorants, which is not the functional group, as we previously expected.

Images can be captured and encoded by three primary colors (RGB) and can be decoded and represented through a screen display or a printer. In analogy, is there a possibility that odor information can be encoded into some forms for data storage, transmission and reproduction? According to the achievements of chemical, biological and psychological research, two odorant characteristics may be taken into account:

1. concentrations of odorants,
2. descriptors based on odor qualities.

The threshold of an odor is a significant feature of odor concentration and is normally defined as the detectable value with a probability of 0.5% at which 50% of a group of panelists detect and identify the presence of the odor [87]. Higher odorant concentrations lead to the activation of more glomeruli [88]. Therefore, odorant concentration is a significant property for odor reproduction. In physiology, the term odor detection threshold depicts the lowest concentration of a certain odor compound that is perceivable by the human sense of smell. The term intensity of an odor depicts multiples of threshold concentrations. Rossiter concluded that there are two sensory odor properties in aroma chemicals: intensity and quality [24]. The intensity of an odor is possibly determined by chemical profiles and polar functional groups of chemical compounds. As illustrated in previous paragraphs, there already have some efforts for the quantitative analysis of concentration by means of electronic noses with an array of gas sensors. In recent years, a novel type of gas sensing device, the bioelectronic nose, has been developed, and it offers us another solution for the concentration characterization of odorants [89,90].

A group of chemical compounds that consists of the same carbon chain with the same functional group attached at different positions activates olfactory sensory neuron patterns with remarkable crossover effects [75]. However, humans are still able to discriminate such a group of odorants. Supposing a large array of gas sensors, the sensors of which are carefully selected, is there any possibility to mimic olfactory response to a certain extent? Is there any possibility to discriminate odor qualities rather than the target objects? Supposing that an odor classification is successful for such a quantity of odors, another odor that is close to the odor detected by a sensory system is able to be used in odor reproduction to deceive human noses. In this hypothesis, such a classification should be based on odor qualities, rather than chemical features.

The controversy is still ongoing surrounding the two theories of stereochemistry and vibration, though neither theory is capable of describing the odor-structure relationship to a certain extent. Regardless of the exceptions of ingredients that have similar structures but different odor qualities, odors with similar odor qualities can be categorized into the same clusters and be used in odor reproductions. An odor reproduction system uses aromas that match those descriptors encoded by a sensory system and generated with the quantities calculated from the concentrations. Yamanaka et al. developed an odor recorder with a gas sensor array and odor blender [91]. The pattern from the response of the sensor array was calculated, and aromas that the pattern was matched to were used and blended. The system consisted of a feedback loop for a better map from the pattern to the aroma. T. Nakamoto and K. Murakami proposed a method for odor reproduction using a mass spectrum database [92] in 2009. A target odor mixture was a combination of a list of odor components with their ratio in this mixture, and the target odor was compared with the dataset by mass spectrometry. According to the slow and complicated procedure of detecting odors by mass spectrometers, it is impossible to use this in a real-time odor sensing circumstance.

A large number of aroma sources are used when producing a typical scent in the art of perfumery. However, some aroma sources are used for the purpose of stabilization of the perfume, and other aroma sources are used for strengthening the scent. Hence, a simplification of the odor reproduction recipe should be considered through the elimination of aroma sources that are used for stabilizing and strengthening scents. With the aim of reducing the number of aroma sources used in an odor display, A. Nambu et al. proposed an olfactory map based on the olfactory sensation according to the odor perception evaluation by a number of people [93]. Over 10,000 identified aroma compounds that can be used for producing food scents were simplified to 230 “key food odorants” [94].

## 5. Conclusions

General odor characterizations and odor reproductions have been reviewed in this paper. Owing to the ambiguousness of the olfactory perception mechanism, the development of odor characterizations and odor reproductions is further behind visual and auditory recording and representing technologies. Currently, there is not a theory for odor characterizations, such that an odor sensing system is incapable of recording odor features in some forms. Although there are a number of efforts in the designs of olfactory displays, the deficiency of general odor characteristics leads to the lack of a method for odor reproduction. Nevertheless, more and more evidence has been found in physiology, and odor reproductions with odor characteristics in some form will be executable in the future.

## Figures and Tables

**Table 1:** Summary of gas/odor concentration estimation research works. QMVLR, quadratic multivariate logarithmic regression; TD-SVR, tapped delay lines and support vector machine.

Refs.	E-Nose	Sensor Type	Sensors Used	Chemicals Analyzed	Methods	Field
[29]	unknown	Polymer	20	Acetic acid, propanoic acid, 2-methylpropanoic acid, butanoic acid, 3-methylbutanoic acid, pentanoic acid, phenol, 4-methylphenol, indole, 3-methylindole	liner regression	Food Production
[30]	OdourMapper, AromaScan	Polymer, Polymer	20, 32	Acetic acid, propanoic acid, 2-methylpropanoic acid, butanoic acid, 3-methyl butanoic, 2-methyl butanoic acid, pentanoic acid, phenol, 4-methylphenol, indole, 3-methylindole	liner regression	Food Production
[31]	AromaScan	unknown	32	288 odor samples fromfour pig productionsites and 192 samplesre-used by changinghumidity	ANN	Sci-Research
[32]	EM	MOX	7	CH${}_{3}$OH, C${}_{2}$H${}_{5}$OH, CH${}_{3}$COCH${}_{3}$, CH${}_{3}$COOCH${}_{2}$CH${}_{3}$	ANN	Sci-Research
[33,34]	EM	MOX	38	H${}_{2}$S, NO${}_{2}$	DFA	Environment
[35]	EM	MOX	16	Ethanol, ethyl acetate, ethyl caproate, ethyllactate	MVLR, QMVLR, MLP, SVM	Sci-Research
[36]	EM	Hybrid	5	NH${}_{3}$, NO${}_{2}$	TD-SVR, BPNs, TDNN	Sci-Research
[37]	AromaScan, Alpha M.O.S., EM	Polymer, MOX, MOX	32, 12, 6	CH${}_{3}$COCH${}_{3}$, C${}_{2}$H${}_{5}$SH	Linear regression, ANN	Sci-Research
[38]	Single sensor, EM	MOX, MOX	1, 11	13 chemicals (not mentioned)	Gaussian process regression	Sci-Research
[39]	Alpha M.O.S.	MOX	18	Phenol	ANNs, hybrid models	Sci-Research
[27,40]	EM	MOX	6	Ethanol, Acetone, Limonene, H${}_{2}$S, Trimethylamine	PCA	Environment

**Table 2:** Performance comparison of olfactory displays. Resp., response; PC, power consumption; NOs, number of odors; SAW, surface acoustic wave.

Refs.	Type	Resp. Rate	Noise	PC	NOs	Forms	Complexity
[45]	Thermalization	Low	Very low	High	Medium	Projected	Low
[51,52]	Ink-jet	High	Low	High	High	Projected	High
[53]	Spray	High	Low	High	Low	Projected	High
[46]	Air blowing	Low	Low	Low	Low	Projected	Low
[56]	SAW	High	Very low	High	Medium	Projected	Low
[47,48]	Air blowing	Low	Low	Low	Low	Projected	Low
[59]	Piezoelectrical	High	Low	Low	Low	Projected	Low
[57,58]	SAW	High	Very low	Low	Low	Wearable	Medium
[60]	Piezoelectrical	High	Low	Low	Medium	Projected	Low
[50]	Thermalization	Low	Low	Low	Low	Wearable	Low
[49]	Thermalization	Low	Low	High	Low	Projected	High
[61]	SAW	Low	High	Low	Low	Projected	Low
